# Tradition in Translation: Cross-Cultural Analysis of Meditative Practices in Psychiatric Care

**DOI:** 10.1192/j.eurpsy.2026.11893

**Published:** 2026-07-31

**Authors:** J. B. French, L. Schmidt, B. R. Carr

**Affiliations:** 1 University of Florida College of Medicine, Gainesville, FL, United States; 2 Department of Psychiatry, University of Florida College of Medicine, Gainesville, FL, United States

## Abstract

**Introduction:**

Meditation is increasingly integrated into psychiatry, often presented as a culturally neutral intervention. Yet meditative forms emerge from distinct traditions, each framed by particular ontologies, aims, and techniques. When imported into psychiatry, these practices are reinterpreted, sometimes shedding elements central to their original meaning and mechanism. This project examines how cultural origins shape both the function and the ethical reception of meditation in psychiatric contexts.

**Objectives:**

To (1) compare selected meditative traditions by cultural-philosophical origin, (2) assess how these are modified in clinical adaptation, and (3) identify ethical and clinical issues when culturally embedded practices are reframed as therapy.

**Methods:**

A targeted literature review in cultural psychiatry, anthropology of religion, and clinical psychology, supplemented by reference mining of key ethnographic and phenomenological studies. Analytical comparison considered intentionality, ritual form, and epistemic authority in source traditions, and clinical modifications noted in psychiatric literature.

**Results:**

Traditions differ in their logic of change: some focus on cultivating insight into impermanence and selfhood; others employ symbolic visualization or repetitive sound for spiritual alignment. In psychiatric adaptation, these are typically reframed in physiological or attentional terms, omitting metaphysical context. While this can increase accessibility, it may also alter efficacy or generate dissonance when patients expect spiritual depth. Ethical concerns arise when origins are obscured or selectively acknowledged, especially with patients for whom religious identity is clinically relevant.

**Conclusions:**

Meditation in psychiatry is not a single, context-free method but a family of culturally rooted practices. Responsible integration requires attention to both mechanism and meaning, and transparent dialogue with patients about origins, aims, and possible cultural tensions.

**Disclosure of Interest:**

None Declared

